# Cardiorespiratory optimization during improvised singing and toning

**DOI:** 10.1038/s41598-017-07171-2

**Published:** 2017-08-14

**Authors:** N. F. Bernardi, S. Snow, I. Peretz, H. D. Orozco Perez, N. Sabet-Kassouf, A. Lehmann

**Affiliations:** 1 Laboratory for Brain, Music and Sound Research (BRAMS), Montreal, Canada; 2 Department of Psychology, McGill University, Montreal, Canada; 3 Centre for the Arts in Human Development, Concordia University, Montreal, Canada; 4 Department of Psychology, University of Montreal, Montreal, Canada; 5 Centre for Research on Brain, Language and Music (CRBLM), Montreal, Canada; 6 McMaster University, Department of Psychology, Neuroscience and Behavior, Hamilton, Canada; 7 Department of Otolaryngology Head & Neck Surgery, McGill University, Montreal, Canada

## Abstract

We evaluated the effect of different forms of singing on cardiorespiratory physiology, and we aimed at disentangling the role of breathing from that of vocal production. Cardiorespiratory recordings were obtained from 20 healthy adults at rest and during: a) singing of familiar slow songs as in the standard form of Western culture; b) improvised vocalization of free vowel sounds, known as *toning*. To disentangle the role of breathing from that of vocal production, we compared the vocal conditions with matched breathing-only conditions. Toning significantly improved heart rate variability, ventilatory efficiency and slowed respiration to almost exactly six breaths per minute (p < 0.001), a pattern that is known to optimize cardiovascular function and that coincides with the period of endogenous circulatory rhythms. Singing songs also positively impacted cardiorespiratory function, although to a lesser extent. The breathing pattern imposed upon participants in the absence of vocal production was sufficient to generate the physiological benefits. The effects of toning are similar to what has been previously described as a result of engaging in formal breathing exercises. Toning and singing may offer an engaging and cost effective tool to trigger beneficial respiratory patterns and the related cardiovascular benefits.

## Introduction

Singing is being increasingly considered as a tool to support rehabilitation of various medical and psychological conditions, for its potential to provide a low-cost, social and highly engaging training of respiratory function^1^. While the psychological benefits of singing have received significant empirical support^2^, it is less clear if, and by which physiological mechanisms, singing may favourably impact cardiorespiratory function *per se* (see ref. 3 for a review). Part of this ambiguity derives from the fact that the term *singing* refers to a number of practices that differ substantially from each other, with operatic, country-pop and religious forms being only a few examples of this wide spectrum. The fact that singing mostly happens in social contexts further complicates an objective assessment of the effects of singing itself. When singing in a group, several other factors may be at play, from low-level motor, respiratory and vocal contagion within the group^4^ to higher-level social cohesion^5^. Adding to this complexity, breathing is also altered during singing. This means that the physiological changes observed during vocal production could be due to singing or to the respiratory manipulation alone, or to the combination of the two.

A few recent studies have investigated the cardiorespiratory changes during singing. For example, Vickhoff *et al*.^6^ showed that choir group singing improves heart rate variability (HRV) and promotes inter-individual synchronization of HRV fluctuations. The interest of these results lies in the fact that HRV is a significant predictor of cardiovascular health. Reduced HRV has been associated with conditions such as hypertension^7^, heart failure^8^ and work stress^9^. Conversely, increased heart rate variability, within the normal range, is believed to reflect a greater capacity of the body to dynamically adjust to the ever-changing internal and external environment, and to reflect a healthy interplay between the sympathetic and parasympathetic branches of the autonomic nervous system^10^. Vickhoff *et al*.^6^ also showed that the effects of singing are dependent on the specific form of singing, with mantra sung at a rhythm of 6 breaths/minute (0.1 Hz) yielding the maximum increase in HRV and inter-individual synchronization. In another choir study, Olsson *et al*.^11^ showed that group singing of slow songs resulted in slower heart rate and greater HRV compared to faster songs. Interestingly, these authors also compared singing with silent breathing at 0.1 Hz, showing that the latter induced the slowest heart rate and highest HRV.

The aim of the present study was two-fold. In the first place, we investigated the cardiorespiratory effects of engaging in toning, a form of improvisation-based open vowel vocalization. The previous studies described above have illustrated that not all forms of singing equally impact the cardiorespiratory system. The nature of the music sung, such as its tempo and phrases, emerged as a key element in mediating the benefits of singing. Because previous studies used pre-determined songs, it is not known whether favourable changes to cardiorespiratory function might be observed as a result of singing in one if its most accessible forms, that is, the improvised vocalizations of open vowel sounds performed by a single and untrained individual.

In the second place, we aimed to establish whether the potential benefits associated with singing could be attributed to the vocal production, or to the respiratory manipulation, or to the combination of the two. Breathing has been consistently shown to be a major actor in determining the physiological changes associated with singing. For example, Vickhoff *et al*.^6^ observed in their choir study that the musical structure determined respiration rate, with a cascade effect in the HRV. Here, we contrasted cardiorespiratory function during singing with a non-vocal condition in which the respiratory patterns were matched to what participants had themselves produced during the vocal conditions.

In the present study we contrasted two different forms of vocalization. In a *song* condition, participants sang familiar slow songs of their preference. This condition was meant to gauge the effects of singing when using the rhythmic and melodic forms that are familiar to Western individuals. In a *toning* condition, participants voiced the length of the exhalation on a freely chosen pitch using open vowel sounds^12^. This form of vocalizing is improvised, in the sense that participants can change the pitch and vowel sound used for each exhalation, as they choose, or repeat the same one if they prefer. We chose toning as a representative exemplar of vocal practices that, rather than generating a predetermined rhythmic and melodic structure, invites a state of moment-by-moment, non-judgmental awareness, a key feature of several meditative practices^13^. In both Eastern and Western traditions, forms of meditative vocal production such as the chanting of prayers and mantras have been found to employ a breathing pattern with a frequency of 0.1 Hz^14^. This respiratory dynamic is of interest as it maximizes the beneficial effect of respiratory modulations on cardiovascular function^15^. These forms of ritual chanting are geared towards meditation, and impose the 0.1 Hz breathing frequency on the singer, by means of repetition of certain sentences and/or by the turn-taking in the group recitation dynamic. Toning offers a freer, less structured model of vocalization, which can shed light on the origins of meditative forms of vocal production. In fact, by studying individual participants engaged in toning, we could ask whether breathing frequencies close to 0.1 Hz might occur spontaneously, even in non-trained individuals, as a result of producing free vocal sounds within a mindfulness framework.

Well aware of the importance of the social context in natural singing, here we deliberately studied isolated participants, and we asked them to engage in singing. The purpose of this was to establish the effects of singing in the absence of other kind of external influences. We only studied participants without previous singing training, as the value of singing for clinical purposes requires its benefits to be accessible to the general population.

## Results

### Respiratory frequency

**Figure 1:**
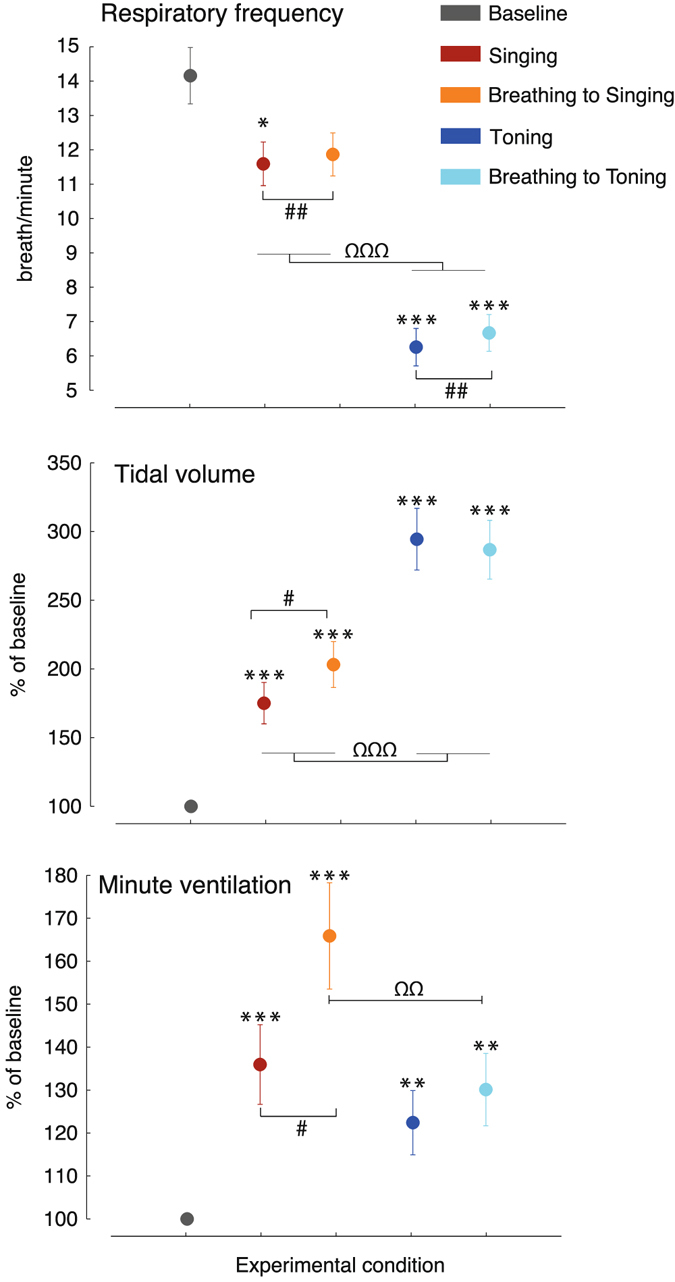
Respiratory data. Respiration slows down relative to baseline during song singing and even more so during toning, reaching ~6 breaths/minute (0.1 Hz). Singing is accompanied by an increase in both tidal volume and minute ventilation relative to baseline. Toning accentuate even further the increase in tidal volume while only moderately increasing ventilation compared to baseline, suggesting respiratory optimization. * Statistically significant difference compared to baseline; # significant difference between the vocal and the matched paced breathing condition; Ω significant difference between song singing and toning. ^*, #, Ω^p < 0.05; ^**, ##, ΩΩ^p < 0.01; ^***, ###, ΩΩΩ^p < 0.001. Bars indicate mean ± SEM.

Figure 1 shows the results for the respiratory variables. Baseline respiratory frequency was 14.1 breaths/minute. During toning, respiratory frequency dropped to 6.2 breaths/minute, significantly slower than baseline (p < 0.001). Breathing rate during singing was 11.6 breaths/minute, significantly faster than toning (main effect of Vocal type: F_(1,19)_ = 78.3, p < 0.001, η^2^_p_ = 0.81) but still slower than baseline (p = 0.03).

### Tidal volume

Tidal volume increased in all conditions compared to baseline (all p < 0.001). The increase observed during toning and toning-like silent breathing was significantly greater than during singing and singing-like silent breathing (Task by Vocal type: F_(1,19)_ = 10.2, p = 0.005, η^2^_p_ = 0.35; post-hoc comparisons: all p < 0.001). Tidal volume was higher during singing-like silent breathing compared to singing (p = 0.034), whereas there were no differences between toning and toning-like silent breathing.

### Minute ventilation

Minute ventilation increased in all conditions compared to baseline (all p < 0.01). Minute ventilation was statistically comparable between the two vocal productions (p = 0.2), showing a trend for lower ventilation during toning compared to singing. This is striking when considering that tidal volume was significantly higher during toning, compared to singing. Paced breathing matched to singing prompted significantly higher minute ventilation, compared to both singing (Task by Vocal type interaction: F_(1,19)_ = 9.7, p = 0.006, η^2^_p_ = 0.34, post-hoc: p = 0.013) and paced breathing to toning (p = 0.008).

### Heart rate and Heart rate variability

**Figure 2:**
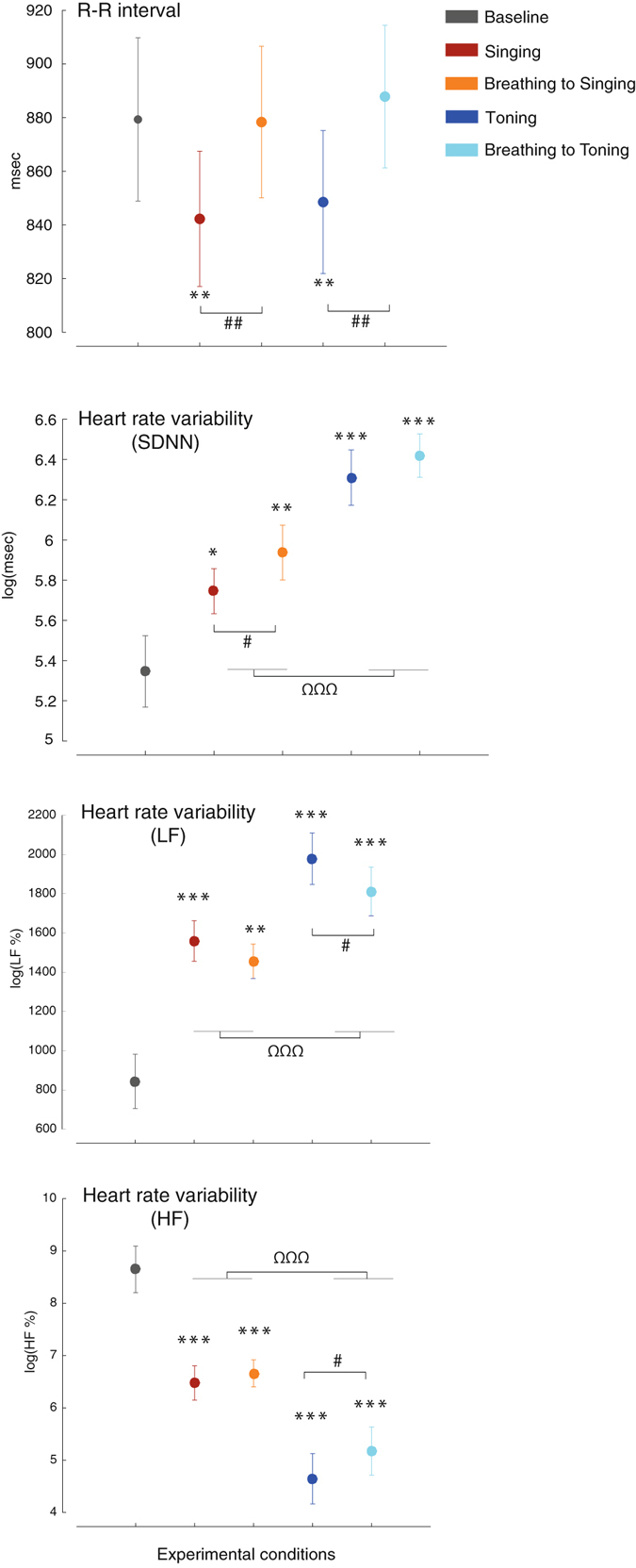
Cardiovascular data. Heart rate increases during vocal production, similarly for song singing and toning. Overall heart rate variability (SDNN) increases in all conditions relative to baseline, significantly more during toning compared to singing, and significantly more during silent breathing compared to the matched vocal conditions. For LF and HF heart rate variability see text. All conventions as in Fig. 1.

Figure 2 shows the results for the cardiovascular variables. Heart rate was faster during vocal productions compared to baseline and paced breathing, with no differences between toning and singing (main effect of Task: F_(1,19)_ = 13.0, p = 0.002, η^2^_p_ = 0.41). Time-domain HRV (SDNN) increased in all conditions compared to baseline, and significantly more during toning compared to singing (main effect of Vocal type: F_(1,19)_ = 43.4, p < 0.001, η^2^_p_ = 0.70). Time-domain HRV also increased significantly more during the silent breathing conditions, compared to their sung counterpart (main effect of Task: F_(1,19)_ = 4.7, p = 0.043, η^2^_p_ = 0.20; the interaction Task by Vocal type was not significant: F_(1,19)_ = 1.3, p = 0.27). The frequency-domain analyses of HRV showed an increase LF power when singing or breathing to singing compared to baseline (p < 0.001 and p = 0.002, respectively), and an even greater increase when toning or breathing to toning compared to both baseline (both p < 0.001) and singing (Main effect of Vocal type: F_(1,19)_ = 17.7, p < 0.001, η^2^_p_ = 0.48). Vocalizing prompted a greater increase in the LF compared to matched silent breathing (main effect of Task: F_(1,19)_ = 8.3, p = 0.009, η^2^_p_ = 0.31; the interaction Vocal type by Task was not significant). The analysis of the HF provided the exact mirror image of the LF. The HF power decreased during singing and breathing to singing compared to baseline (both p < 0.001), and decreased even further during toning (main effect of Vocal type: F_(1,19)_ = 25.4, p < 0.001, η^2^_p_ = 0.57). Reversing the pattern observed in the LF, the HF power was higher during the breathing conditions compared to the vocal conditions (main effect of Task: F_(1,19)_ = 5.1, p = 0.036, η^2^_p_ = 0.21; the interaction Vocal type by Task was not significant).

## Discussion

Improvised open vowel vocal production during the toning condition triggered a breathing frequency of ~0.1 Hz. This was significantly slower compared to baseline breathing rate and compared to breathing during standard song singing. Breathing during toning was also deeper, showing a robust increase in tidal volume with only moderately increased minute ventilation. Furthermore, toning was associated with increased heart rate variability. These elements together depict a pattern of cardio-respiratory optimization as a result of engaging in toning. These changes were similarly observed during the non-vocal condition matched for breathing pattern during toning. This points to the breathing, rather than to the sound production *per se*, as the key factor for mediating the observed physiological effects.

### Slow deep breathing

The respiratory pattern generated during toning, characterized by a rate of about 0.1 Hz and a markedly increased tidal ventilation with only a moderate increase in minute ventilation, remarkably coincides with a well-known breathing manipulation, often referred to as “slow deep breathing” (DB). This observation is of clinical relevance, as this breathing pattern has been previously shown to have beneficial effects through a cascade of physiological mechanisms. In the first place, DB is known to increase effective alveolar ventilation, as it increases oxygen saturation with minimal or no additional ventilatory work or hyperventilation^16–19^. This likely happens because the increased depth of breathing reduces the ventilation/perfusion mismatch (physiological dead space), as it provides a better perfusion of the upper lung areas and a better ventilation of the lower lung areas. The total gas exchange area is also increased, as more alveoli are recruited. Optimizing alveolar ventilation has the consequence of reducing the need for ventilatory compensations to changes in gas composition (chemoreflex response). For example, the increase in ventilation due to hypoxia is substantially lowered if participants are using DB, indicating a decreased urge to breath despite the respiratory challenge^20^. The effects of DB are not limited to the respiratory system. By increasing cardiac baroreflex^18, 21^, the repeated practice of DB may significantly reduce blood pressure^22^. DB also reduces markers of sympathetic activations like muscle sympathetic nerve activity^23^ and arterial stiffness^24^. On the other hand, DB strengthens vagal modulations, as evidenced by increased respiratory sinus arrhythmia and increased HRV^15^, as also reflected in our results, and augmented spontaneous fluctuations in blood pressure^14^. Consistently with these physiological changes, DB has been shown to increase the subjective feeling of relaxation and to decrease anxiety^25^.

### Toning, singing and breathing

In the literature as well in clinical practice, DB is typically imposed on the participants, mostly through auditory cues^26^ or by reciting specific verbal sequences^14^. Here we show that a similar breathing pattern develops spontaneously in healthy individuals as they engage in toning, i.e. as they produce free open vowel sounds within a mindfulness framework. Mindfulness possibly represents the pivotal connection between the toning we investigated here and the ritualistic formulas utilized in previous research. In fact, the origin of those previously described chants is also to be found in forms of awareness and spiritual practices. Examples of this are the practice of the full Yogic breathing^20^ and the recitation of Eastern (Mantras) and Western (Ave Maria) prayers^14^. We speculate that different mind-body traditions throughout history might have converged in constructing their practices around the basic mechanism of DB. DB would serve in this sense as a simple but effective way to optimize cardio-respiratory function, which in turns would promote an optimal sympathetic-parasympathetic balance, providing the optimal state for pursuing various spiritual, emotional and social goals. Central to this speculation is the fact, documented here for the first time, that this self-regulatory breathing dynamic can emerge spontaneously. In fact, breathing deeply at about 0.1 Hz appears as something that anybody could readily connect to, and benefit from. Our study provides useful insights as to what some conditions that favor the emergence of this breathing pattern could be. Toning, i.e. voicing sounds on the exhalation within a moment-to-moment awareness, appears in our results to be the trigger of the DB pattern. In fact, spontaneous breathing in the absence of toning, as recorded during the baseline condition, was faster and shallower, in the range expected for healthy non-trained individuals. Singing slow songs following a pre-determined melodic-rhythmic pattern also promoted a positive physiological change in terms of a slower and deeper breathing pattern, in addition to an increase in HRV, compared to baseline. However, these changes were smaller than observed during toning, describing altogether a pattern different from DB.

On the other hand, our data clarify that the breathing pattern, even when imposed upon participants and in the absence of vocal production, is sufficient to generate the physiological benefits. In particular, there was no difference between toning and the matched breathing condition in terms of tidal volume and minute ventilation. HRV variability increased in all conditions compared to baseline, suggesting a favourable increase in the parasympathetic activity. Silent breathing proved to be even more favourable than the matched vocal conditions, prompting a stronger increase in HRV. This observation, together with the slower heart rate and the stronger HF-HRV observed when silently breathing, suggests that singing or toning may provide a mild form of sympathetic stimulation, compared to the more neutral breathing condition. Our findings are consistent with previous observations that silent slow breathing yields effects on HRV that are at least comparable, and at times superior, to singing traditional songs^11^. These finding are also consistent with previous studies in which it was suggested that the change in breathing pattern could be the key element mediating the effects of musical structure on HRV^6^. It would be interesting in future research to investigate whether paced breathing and toning may differ in terms of the participants’ subjective experience, emotional engagement and possibly their outcomes on stress and well-being. It should be also noted that the vocal production might offer a protection against the risk of hyperventilation associated with paced breathing, especially at faster rates. In this respect, we observed significantly higher minute ventilation during breathing to singing, compared to singing, despite a closely matched respiratory frequency and tidal volume.

### Limitations

A limitation to be aware of in the present study pertains to the influence that the recorded instructions might have had on the participants’ respiratory and vocal behaviour. The breathing frequency in the recorded examples was slower for the toning (4.6 breaths/minute) compared to the singing (16.2 breaths/minute) condition. Thus, to some extent participants might have slowed their breathing during toning in an attempt to imitate the examples provided, rather than generating a genuinely spontaneous vocal production. The reason why we presented examples in the first place was to illustrate to the study participants what was required from them since they were unfamiliar with what toning was, and it would have been difficult to conduct an experiment without some demonstration. We also note that, whereas listening to the instructions might have had an influence on the rate of breathing, this influence may not extend to the ventilatory pattern, which cannot be inferred by listening to the sound of another person breathing or singing. Furthermore, the breathing rate during the recorded example for singing was faster than the average baseline breathing rate. If participants were merely being influenced by the instructions, one would have expected a breathing rate during singing similar to, or faster than, baseline. However, the average breathing rate during singing was significantly slower than baseline. As a general approach, previous studies provided auditory cues to have participants maintain the prescribed breathing rate during singing (e.g., metronome, piano accompaniment). Conversely, in the present study the vocal production was generated in the absence of any of such cues, reinforcing the idea of a spontaneous element in the behaviour described here. Another limitation worth considering is the artificiality and constraints of the situation in which participants were asked to sing. On the other hand, the fact that despite these constraints we could still observe reliable cardiorespiratory modifications suggests that these effects are robust, and could be expected to be even stronger in a more naturalistic situation. Another limitation that should be considered is the slight (0.3 breaths/minute) but statistically significant mismatch between the vocal and the breathing conditions, which could be improved for future research. Furthermore, the method used to assess ventilation was not quantitative, so these data could be analyzed only in terms of changes with respect to the baseline. On the other hand, the use of a facemask or a mouthpiece would have substantially interfered with the vocal production, in addition to alter baseline ventilation parameters, as previously shown^27^.

In conclusion, improvised vocal production in the form of toning triggered breathing at ~0.1 Hz, a pattern that is known to optimize cardiovascular function and that coincides with the period of endogenous circulatory rhythms. Toning appears as a cost effective, safe and potentially rewarding way to trigger beneficial respiratory patterns and the related cardiovascular benefits.

## Methods

### Participants

We tested 20 participants without cardiovascular or respiratory medical conditions and without prior singing training (age, M ± SD: 24.2 ± 3.8; 14 female; 2 additional participants were not included in the final sample as they failed to comply with the instructions for the silent breathing condition, showing a mismatch in respiratory rate between the vocal and the silent condition >1.96 SD from the mean group mismatch, see below). All participants gave written informed consent to participate in the study, which was approved by the Human Research Ethics Committee of Concordia University (Certification number: 30004786). All aspects of the study were conducted in accordance to the principles laid out in the Declaration of Helsinki.

### Procedure

Measurements from each participant were obtained through a portable custom unit that was capable of obtaining: i) electrocardiogram, from 3 standard thoracic leads, and ii) respiratory excursions from the abdomen and from the chest by inductive plethysmography (see ref. 4 for further details). Participants were tested in a 2-hr session comprising: a) resting baseline, b) singing slow songs freely chosen by the participants themselves, c) toning, d) silent breathing paced by a visual cue matched to the breathing pattern generated by the participant during singing songs (see below), and e) silent breathing matched to the respiratory pattern generated during toning. Each condition lasted 7 minutes. The order of presentation of conditions b) and c) was randomized across participants, and this order was replicated for the presentation of condition d) and e). The study was part of a larger project which also involved brain electrophysiological (EEG) recording, standardized emotions questionnaires and semi-structured interviews, the results of which are reported elsewhere. To increase the homogeneity of the two vocal conditions, in the song condition participants were instructed to hum or sing syllables ending in a vowel sound such as: “La la la”, rather than sing the actual words to the song. Participants were tested with their eyes open, seated on a comfortable chair. Prior to each condition, participants received standardized instructions through a pre-recorded audio file (the standardized instructions and examples are available at the online repository: https://soundcloud.com/nicol-francesco-bernardi/sets/cardiorespiratory-optimization-during-improvised-singing-and-toning). The breathing frequency in the recorded examples was 4.6 and 16.2 breaths/minute for the toning and singing conditions, respectively. During baseline, toning and singing participants maintained their gaze on an “x” sign, surrounded by a static circle, presented on a computer screen ~80 cm facing them. During the silent breathing conditions, the circle was programmed to concentrically expand and shrink according to the breathing pattern generated by the same participant during the song or toning condition. In this case, participants were instructed to inhale and exhale in synchrony with the expansion and contraction of the circle, respectively, and to regulate the volume of their breathing proportionally to the size of the circle. Participants were not told that the animated circle corresponded to their own breathing pattern. To control the rate and the amount of change of the animated circle we: 1) summed the signals of the two respiratory bands; 2) applied a low-pass Butterworth filter (cut-off: 1 Hz); 3) normalized the resulting signal between 0 and 1, corresponding to the minimum and maximum respiratory excursion for that participant, and corresponding to a size of the circle on the screen of 1.7 and 13.4 cm, respectively. Using this procedure, we obtained an average mismatch in breathing rate between paced breathing and the corresponding vocal condition of 0.3 breath/min, in the direction of a slightly faster breathing rate during the paced breathing (this difference was statistically significant, p = 0.001).

### Data analysis

We identified the peak of the R wave of the ECG in the electrocardiogram and constructed the series of the heart period by measuring the R-R interval. The analysis of heart rate variability (HRV) was performed using the HRVAS toolbox in Matlab^28^. Time-domain HRV was assessed by computing the standard deviation of the R-R time series (SDNN). Frequency-domain HRV was assessed by calculating the power spectrum density (PSD) in the low frequency (LF, 0.03–0.15 Hz) and high frequency (HF, 0.15–0.6 Hz) bands. The LF band is believed to reflect the combined activity of both the parasympathetic and sympathetic branches of the autonomic nervous systems, whereas the HF band is more selectively influenced by parasympathetic modulations^29^. The power within each band was obtained by integrating the PSD between the band frequency limits. For the purpose of statistical analyses, we utilized the percentage of the sum of the LF and HF amplitude (LF%, HF%). The SDNN, LF% and HF% data were transformed using a Box-Cox transformation to correct for skewed distribution (lambda = 0.15, 1.9 and 0.4, respectively). The analysis of respiratory frequency, tidal volume (i.e., the volume of air displaced during inhalation) and minute ventilation were performed on the sum of the signals obtained from the two belts. A semi-quantitative intra-subject analysis of ventilation was obtained by comparing the relative (percentage) changes in tidal volume and minute ventilation induced by different experimental conditions. Although the device used for the present study does not allow obtaining tidal volume and minute ventilation in absolute values (mL and L/min, respectively), we relied on the strong linear relationship between tidal volume and the inductive belt signals^30^, thus allowing us to obtain ventilation in relative units. In our experiment, the limitation of the semi-quantitative analysis is compensated by the freedom from interference with the vocal production and with spontaneous breathing that would have occurred with mouthpieces^27^. Differential scores relative to baseline were computed for each dependent variable. Changes relative to baseline for each dependent variable were assessed statistically be means of t-test relative to 0 on the differential scores. To statistically compare conditions with each other, we run 2 × 2 ANOVAs on the differential scores using Task (levels: Vocal vs. Breathing) and Vocal type (levels: Song vs. Toning) as repeated measures factors.
